# Cells derived from murine induced pluripotent stem cells (iPSC) by treatment with members of TGF-beta family give rise to osteoblasts differentiation and form bone *in vivo*

**DOI:** 10.1186/1471-2121-13-35

**Published:** 2012-12-15

**Authors:** Feng Li, Christopher Niyibizi

**Affiliations:** 1 Department of Orthopaedics and Rehabilitation, Division of Musculoskeletal Sciences, Pennsylvania State University College of Medicine, H089, 500 University Drive, Hershey, PA, 17033, USA; 2 Department of Biochemistry and Molecular Biology, Division of Musculoskeletal Sciences, Pennsylvania State University College of Medicine, Hershey, PA, USA

**Keywords:** iPSC, Stem cells, Osteoblasts, TGF-beta, Bone formation

## Abstract

**Background:**

Induced pluripotent stem cells (iPSC) are generated by reprogramming somatic cells into embryonic like state (ESC) using defined factors. There is great interest in these cells because of their potential for application in regenerative medicine.

**Results:**

iPSC reprogrammed from murine tail tip fibroblasts were exposed to retinoic acid alone (RA) or in combination with TGF-β1 and 3, basic fibroblast growth factor (bFGF) or bone morphogenetic protein 2 (BMP-2). The resulting cells expressed selected putative mesenchymal stem cells (MSCs) markers; differentiated toward osteoblasts and adipocytic cell lineages *in vitro* at varying degrees. TGF-beta1 and 3 derived-cells possessed higher potential to give rise to osteoblasts than bFGF or BMP-2 derived-cells while BMP-2 derived cells exhibited a higher potential to differentiate toward adipocytic lineage. TGF-β1 in combination with RA derived-cells seeded onto HA/TCP ceramics and implanted in mice deposited typical bone. Immunofluorescence staining for bone specific proteins in cell seeded scaffolds tissue sections confirmed differentiation of the cells into osteoblasts *in vivo*.

**Conclusions:**

The results demonstrate that TGF-beta family of proteins could potentially be used to generate murine iPSC derived-cells with potential for osteoblasts differentiation and bone formation *in vivo* and thus for application in musculoskeletal tissue repair and regeneration.

## Background

Induced pluripotent stem cells (iPSC) have generated hope and excitement because of the potential they possess in regenerative medicine. Since the discovery by Yamanaka and colleagues that somatic cells can be reprogrammed to embryonic like state (ESC), numerous reports have emerged focusing on methods to generate them efficiently and safely for future clinical applications [1-7]. Some progress has been made toward development of techniques for generating safer iPSC; for example; generation of virus free iPSC, thus avoiding potential of viral effects on tumor formation, use of protein factors to reprogram somatic cells and reprogramming without using oncogenic factors [5-11]. In addition, efficiencies in reprogramming somatic cells have improved [12-15]. Although more work is still needed to get iPSC closer to clinical application, it is also critical to begin to understand factors that play a role in directing differentiation of iPSC to various cell lineages prior to clinical application. In this regard, several studies focusing on methods to direct iPSC to specific cell lineages are active areas of investigation [16-19]. These approaches emulate methods developed for directing ESC to various lineages [20-22]. Most studies have focused on directing ESC or iPSC to hematopoietic and or neural cells lineages [16-19]. As a proof of concept, iPSC were generated from a humanized mouse model of sickle cell anemia followed by correction of the sickle cell defect. iPSC with the corrected gene were then directed to hematopoietic cell lineage and given back to the somatic cell donor [18]. This proof of concept demonstrated that it was possible to direct iPSC to hematopoietic lineage efficiently at least for murine iPSC.

Directing human ESC or iPSC to neural lineages has also gained some success [17,20,23,24]; Wernig and colleagues showed that iPS-cell-derived dopaminergic neurons could alleviate the disease phenotype in a rat model of Parkinson’s disease [17]. Differentiation of human embryonic stem cells (hESC) and or human induced pluripotent stem cells (hiPSC) to mesenchymal cell lineage have also been reported [25-28]. iPSC were directed to MSCs differentiation and cells were then sorted based on CD24 and CD105 surface antigen expression. Sorted cells were demonstrated to exhibit MSCs characteristics and gave rise to adipocytes, chondrocytes and osteogenic lineage [25]. Differentiated iPSC were also shown to alleviate ischemia in a mouse model. These data showed that iPSC have potential to give rise to cells of mesenchymal lineage, this approach involved cell sorting which is inefficient for clinical application. In addition, this has been successful in human iPS derived cells. Differences between mouse ESC and iPSC have been noted; methods used for differentiation of human ESC or iPSC to specific lineages may not apply to mouse ESC or iPSC. Directing mouse ESC or iPSC specifically to osteoblasts lineage and bone formation *in vivo* has presented a challenge. Previous reports showed that murine iPSC could be induced to osteoblasts differentiation and bone formation; however; Bilousova et al. used retinoic acid alone for derivation of cells from murine iPSC that formed calcified structures in scaffolds *in vitro* and *in vivo*[29]. Morphology of the derived cells using this approach was however not indicated. Jukes et al indicated that, for murine ESC to make bone *in vivo*, a cartilage template is a necessity [30]. Although this approach demonstrated clear bone formation *in vivo*, it is a multistep process and does not directly generate MSCs like cells from iPS. Nevertheless, this approach clearly demonstrated that murine ESC can be manipulated to make bone *in vivo*.

We report here that cells derived from murine iPSC by treatment with TGF-beta family of proteins specifically TGF-beta 1 and 3 have potential to give rise to cells that display osteoblasts characteristics *in vitro* and *in vivo*.

## Results

**Figure 1:**
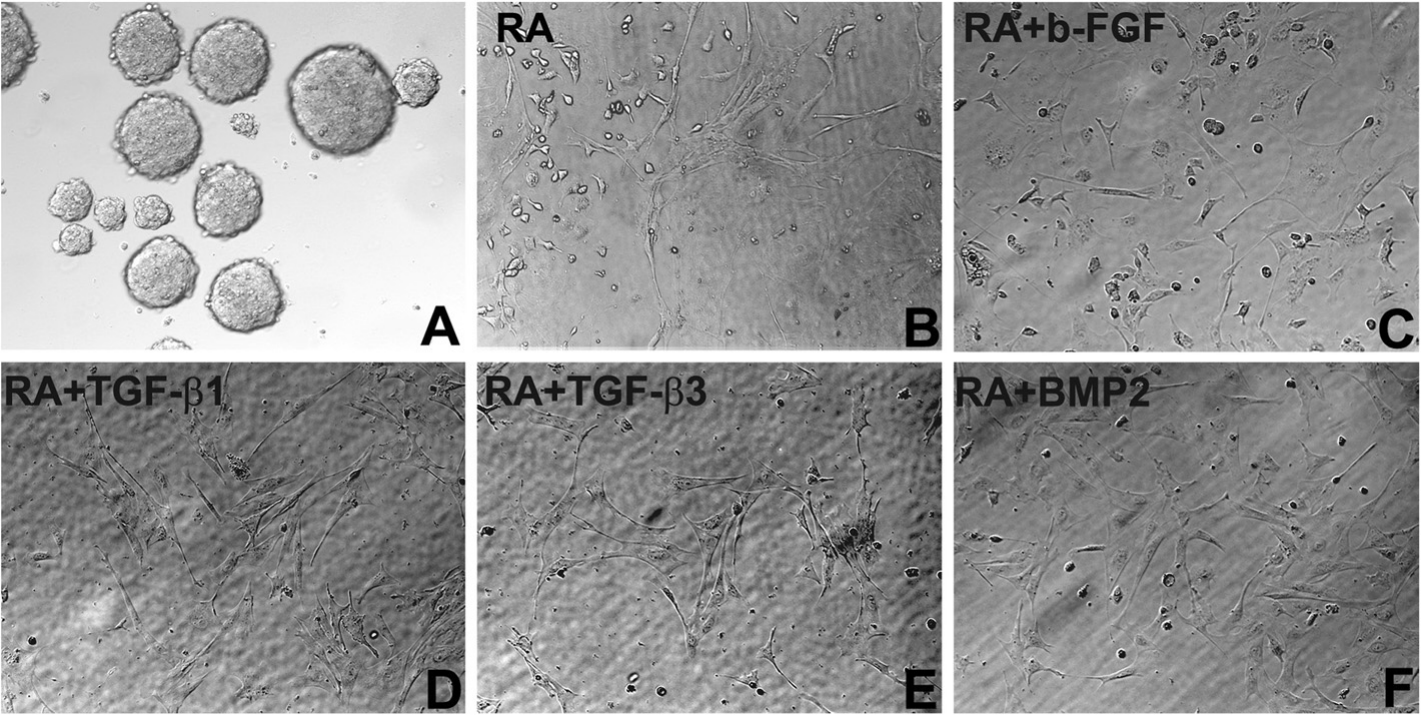
**Embryoid bodies generated from murine iPSC and morphological appearance of iPSC-cells derived by treatment with various growth factors.** (**A**) EBs derived from murine iPSC (**B**) iPSC-cells derived by exposure to RA alone (**C**) iPSC-cells derived by exposure to bFGF and RA (**D**) iPSC-cells derived by exposure to TGF-beta1 and RA (**E**) iPSC-cells derived by exposure to TGF-beta3 and RA (**F**) iPSC-cells derived by exposure to BMP-2 and RA. The round cells within each preparation probably represent undifferentiated cells. Details for generation of each cell population are outlined in the text in materials and methods section. Original magnifications 200X.

Figure 1 shows EBs and morphological appearance of the cells derived from them by incubation in a medium supplemented with b-FGF, TGF-beta1, TGF-beta3 and or BMP-2 in combination with RA or RA alone. The iPSC-derived cells using the above growth factor treatments displayed fibroblastic morphologies (Figure 1). TGF-beta 1 and 3 derived cells contained a population of cells with spindle shaped morphology characteristic of MSCs. Cells derived by RA alone treatment also contained cells with a spindle morphology but there were also more cells with rounded morphology when compared to TGF-beta 1 or 3 population. Cells derived by FGF-RA or BMP-2 comprised cells with flattened morphologies as well as cells with rounded morphologies. Nevertheless all cell populations did not appear homogenous, suggesting that there were more than one cell population present within the preparations. We did not quantitate ratios of subpopulation within each preparation, the goal was to generate cells that were enriched in a population with a higher potential to differentiate toward osteoblastic lineage.

We did not perform gene expression analysis to demonstrate loss of multipotency markers by each of the cell population generated by different treatments. Previously, we demonstrated that cells derived from iPSCs (EBs) by treatment with TGF-beta 1or RA alone lost expression of multipotency markers [31], based on these previous data, it was concluded that these cell populations were devoid of multipotency markers.

**Figure 2:**
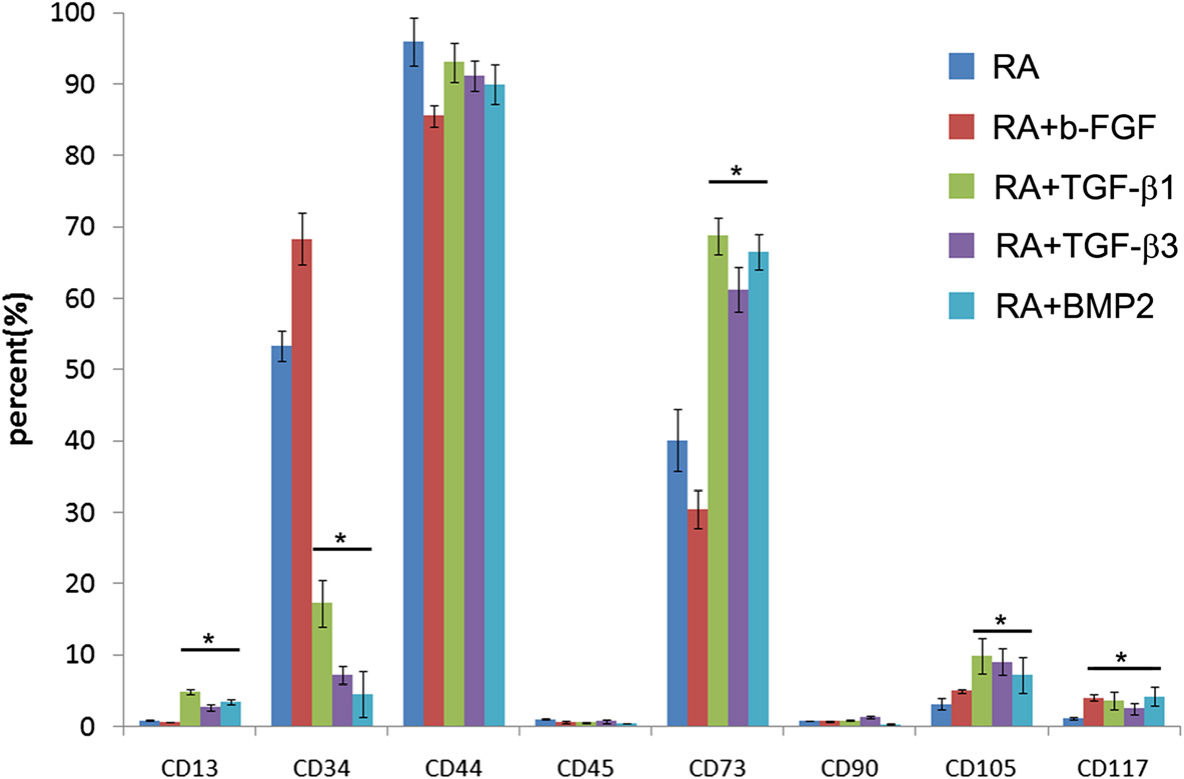
**FACS profile of selected surface antigens by iPSC-cells derived using different factors.** The level of expression for each of the surface antigens that was assessed is indicated in the figure. bFG- derived cells comprise a population expressing high levels of CD34 and CD44. TGF-β1 and 3-derived cells were enriched in populations expressing CD13, CD73 and less CD105. IPSC-cells-derived by exposure to BMP-2 were enriched in a population of cells expressing high levels of CD73 and CD44 but less CD105. P < 0.05.

To further characterize iPSC-derived cells, expression of surface antigens attributed to MSCs was examined by FACS analysis. Expression profiles of the selected surface markers by each of the growth factor derived cells are shown in Figure 2. All cell populations derived by various treatments contained cells that expressed CD13, CD34, CD44, CD73, CD105 and CD 117 at varying degrees. Basic fibroblast growth factor (bFGF) and RA alone derived cells comprised a population expressing high levels of CD34 suggesting that they were enriched in cells of hematopoietic lineage. TGF-beta1 and 3-derived cell populations contained a population of cells expressing high levels of CD73 and less of cells expressing CD34 (Figure 2). All cell populations derived by various treatments, contained a population of cells expressing CD105 at very low levels. In TGF-beta1 and 3 derived cells, only 10% of the cells were CD105 positive. It was not determined whether the CD105 cells were also CD73 positive. CD73 is one of the markers attributed to MSCs and has been used previously to sort cells from hESC with potential to give rise to cells of mesenchymal lineage [32]. Markers expressed by iPSC (EBs) that were not manipulated were not determined, RA derived cells were used as controls in these experiments and this is a common protocol used for ES and iPS cell differentiation. As indicated above these cells expressed lower levels of markers attributed to MSCs when compared to those derived by other treatments except bFGF (Figure 2)*.* Because of lack of specific marker for identifying MSCs; the relationship of CD 73 expressing cells to MSCs could not be verified.

**Figure 3:**
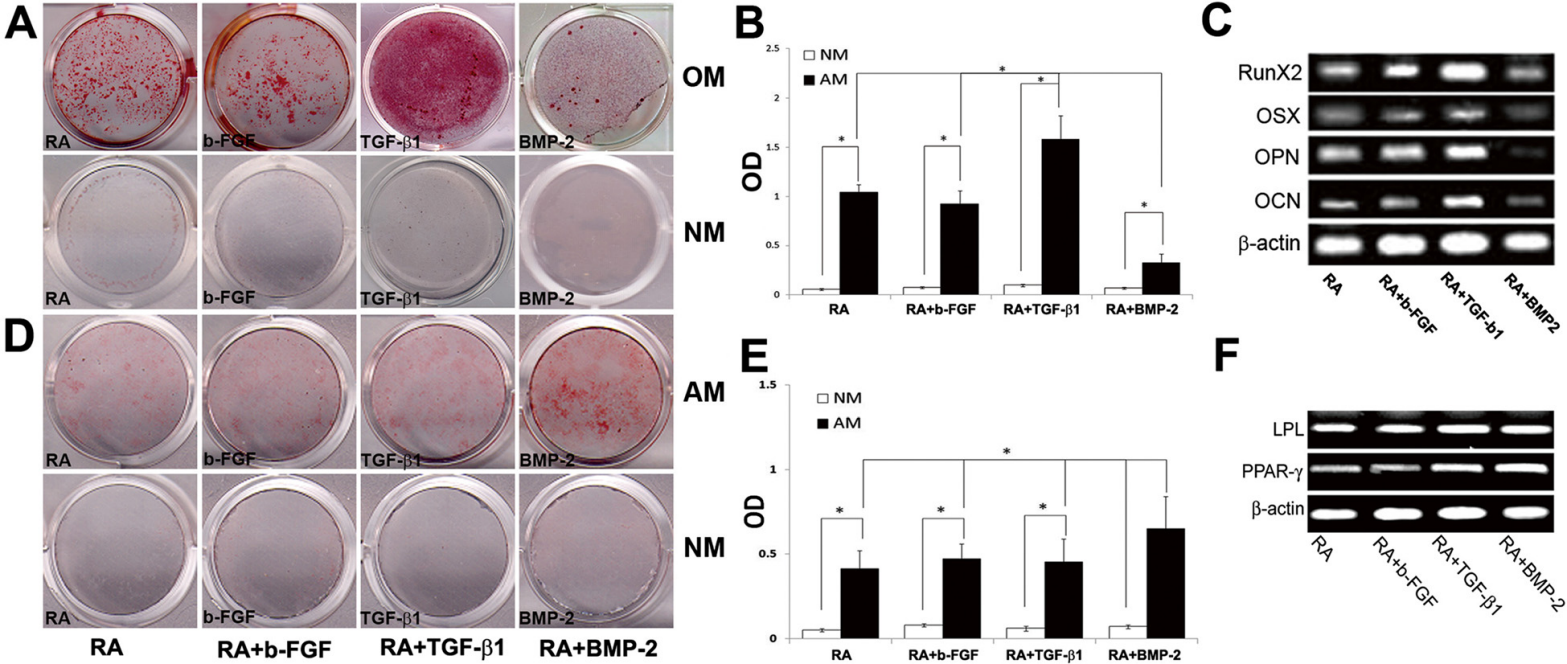
***In vitro *osteogenic, adipogenic differentiation and gene expression of iPSC-cells generated using different factors.** (**A**) Osteogenic differentiation of iPSC-cells-derived by exposure to RA alone; bFGF-RA, TGF-beta1-RA and TGF-beta3-RA. (**B**) quantitation of Alizarin Red deposition by each of the cell population. iPSC-TGF-beta1-RA-derived cells deposited more calcium based on Alizarin Red quantitation. (**C**) Expression of osteoblasts associated genes by differentiating cell populations. iPSC-BMP-2-RA cells express lower levels of osteogenic genes; (**D**) Adipocytic differentiation of iPSC- RA-cells, bFGF-RA-cells, TGF-beta1,3-RA-cells and BMP-2-RA-cells as assessed by Oil Red O staining. (**D**) All cells show adipogenic differentiation but BMP-2 derived cells show higher adipogenesis. (**E**) Quantitation of adipogenic differentiation based on Oil Red O deposition; all cells show adipogenic differentiation but BMP-2 derived cells show higher adipogenesis. (**F**) Semi quantitative PCR of osteoblasts and adipocytes associated genes expression by the iPSC-cells respectively. BMP-2-derived iPSC-cells showed least expression of osteoblasts associated genes but higher adipogenic differentiation markers. P <0.05. NM= normal medium, OM= osteogenic medium, AM= adipogenic medium. OSX= Osterix, OPN= osteopontin, OCN= Osteocalcin.

Next, we examined differentiation of the cell populations toward osteogenic and adipogenic lineages. The results showed that in all treatment groups, the cells were capable of differentiating toward osteogenic lineage but at varying degrees. Retinoic acid alone and or bFG-derived cells exhibited reduced capacity to differentiate toward osteogenic lineage as demonstrated by the quantitation of Alizarin Red deposits in osteogenic differentiating cultures (Figure 3A and B). TGF-beta1- derived cell populations exhibited higher differentiation ability toward osteogenic lineage than the other cell populations generated by other treatments. The cell population derived by treating iPSC with BMP-2 exhibited higher ability to differentiate toward adipogenetic lineage (Figure 3D and E). These data suggest that TGF-beta1 and BMP-2 have distinct activities toward iPSC differentiation. Because iPSC-TGF-beta3-cells had similar differentiation capabilities as TGF-beta1-cells, data is only shown for TGF-beta1 treatment cell populations. Retinoic acid derived cells were used as controls to monitor differences in surface antigen expression by cells generated by various treatments.

Gene expression profile of osteogenic markers confirmed differentiation data. At day 28 following induction of differentiation, all cell populations expressed Runx2, osterix (OSX), osteopontin (OPN) and osteocalcin (OCN) (Figure 3C). Semi quantitative RT-PCR results indicated that BMP-2 derived cells expressed lower levels of osteogenic markers than the cells derived by TGF-beta1; retinoic acid alone and or bFGF cell populations (Figure 3C). In addition, we previously demonstrated that iPSC not treated with supplements did not express osteoblasts related markers [31]*.* These data are in agreement with Alizarin Red staining results shown in Figure 3A and B. Expression of adipocyte related genes confirmed adipogenic differentiation of the cells generated by treatment of iPSC with various growth factors (Figure 3F).

**Figure 4:**
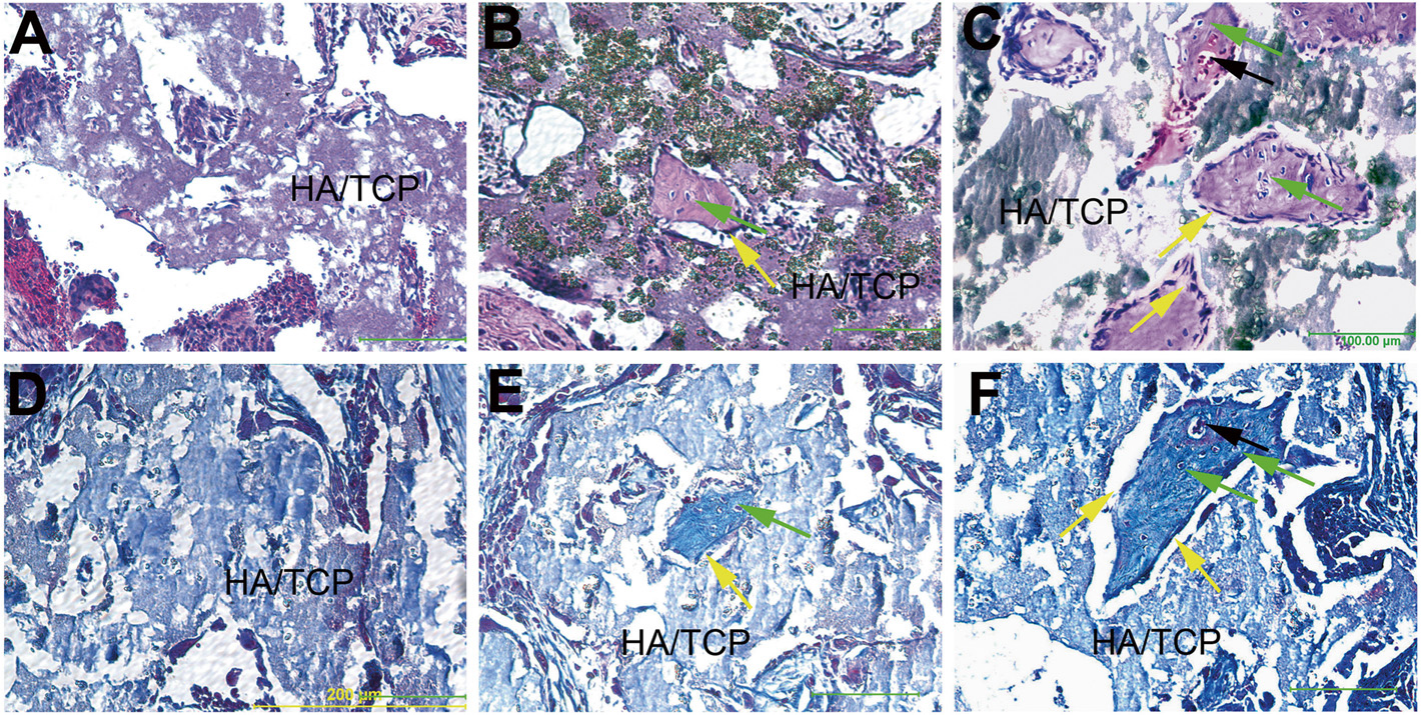
**Histological analysis of bone formation and trichrome staining in ceramic scaffolds seeded with iPSC-TGF-beta1-RA-cell or iPSC-RA-cells and implanted in mice.** (**A**) Tissue section of a ceramic scaffold not seeded with cells; no bone formation is evident, only the ceramic scaffold (HA/TCP) is present. (**B**) Tissue section of a ceramic scaffold seeded with iPSC-RA-cells *shows fewer islands of bone formation scattered in the scaffold.* (**C**) Tissue section of a ceramic scaffold seeded with iPSC-TGF-beta1-RA-cells *show many large islands of bone within the scaffold.* (**D**) Trichrome staining in a tissue section of a ceramic scaffold implanted without cells and stained with trichrome. *There is no staining for collagen, a major protein component of bone indicating absence of bone in this scaffold*. (**E**) Ceramic scaffold implanted with iPSC-RA-cells and stained with trichrome; *staining for collagen (blue) is present within the thin island of bone within the scaffold confirming H and E staining for presence of bone.* (**F**) Ceramic scaffold seeded with iPSC-TGF-beta1-RA-cells. iPSC-TGF-beta1- RA-cells deposited more bone in scaffolds than RA alone derived cells based on histological observations of the bone size within the ceramics. *Trichrome staining in the bone islands is evident confirming presence of bone matrix within the islands*. Osteoblasts lining bone surfaces are indicated by yellow arrows; osteocytes are indicated by green arrows and blood vessels within the newly made bone are indicated by black arrows. *Osteoblasts and osteocytes were identified by their location within bone. Cells lining the surface were presumed to be osteoblasts and cells embedded in bone were assumed to represent osteocytes*. Scale bar 200 μm.

To determine whether the cells had the ability to make bone *in vivo*, they were seeded onto HA/TCP scaffolds and implanted onto the backs of SCID mice. Because cells derived by TGF-beta1 treatment showed higher differentiation ability *in vitro* toward osteogenic lineage, they were assessed for bone formation in ceramics and compared to cells derived by RA alone treatment. Analysis of tissue sections at 5 weeks after implantation, showed that both cell populations were capable of depositing bone in ceramics; however, TGF-beta1 derived cells appeared to deposit more bone with evidence of blood vessels (Figure 4B and C). The newly deposited bone contained surface lining osteoblasts and osteocytes embedded in bone as indicated by yellow and green arrows respectively (Figure 4B,C). HA/TCP ceramics not seeded with cells did not show any bone formation (Figure 4A). These data demonstrated that iPSC derived cells by TGF-β1 treatment exhibit potential to deposit bone *in vivo*.

Collagen deposition by the cells in ceramics was assessed by trichrome staining; HA/TCP ceramics not seeded with cells showed absence of collagen staining as expected (Figure 4D). Ceramics seeded with cell populations from RA alone or in combination with TGF-beta1 treatments revealed staining in areas of bone deposition (Figure 4E,F). Examination of ceramic tissue sections of TGF-beta1 treated iPSC-derived cells indicated presence of more bone than RA treated derived cells (Figure 4E,F). Osteoblasts and osteocytes are indicated by yellow arrows. These data clearly demonstrate that iPSC-derived cells by TGF-beta1 in combination with RA treatment synthesize and deposit organic bone matrix and form bone *in vivo*. From 6 ceramics implanted with the cells, 2 were found to contain few teratomas suggesting that in some cases, there were some residual cells with multipotency within the preparation. Nevertheless, most of the cells derived from iPS by TGF-beta 1 treatment were devoid of cells capable of making teratomas.

**Figure 5:**
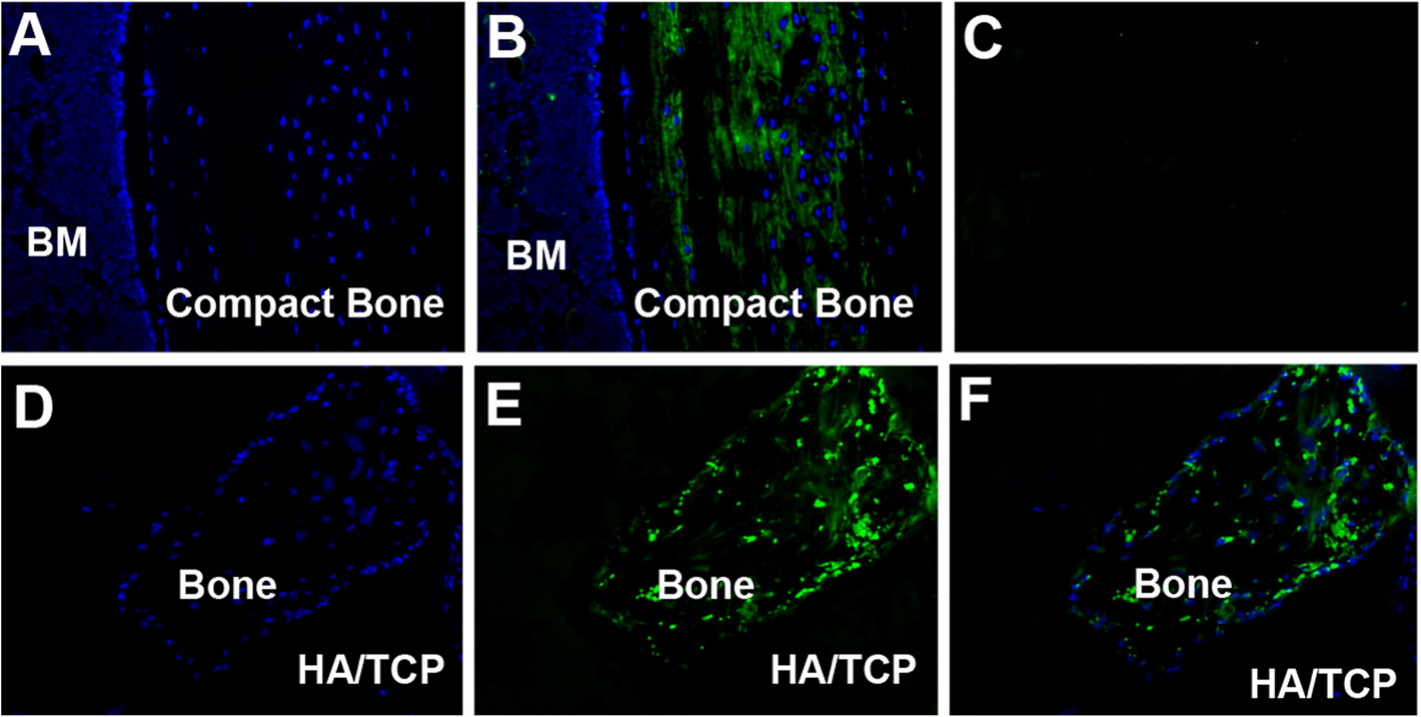
**Immunofluorescence staining for osteocalcin in tissue sections of murine cortical bone and in ceramics seeded with iPSC-TGF-beta1-RA-cells.** (**A**) DAPI staining in a tissue section from a murine cortical bone (**B**) Immunofluorescence staining for osteocalcin in tissue section of cortical bone (**C**) Immunofluorescence staining in a tissue section in which primary antibody was omitted. (**D**) DAPI staining in a tissue section from a ceramic seeded with iPSC-TGF-beta1-RA-cells; (**E**) Immunofluorescence staining of osteocalcin in a tissue section of a ceramic scaffold seeded with iPSC-TGF-beta1-RA-cells; (**F**) Overlay image of D and E showing osteocalcin expressing cells colocalizing with DAPI staining cells. Original magnifications 200X. The data confirm that iPSC-cells derived by TGF-beta1 in combination with RA differentiated into osteoblats *in vivo* and deposited bone.

To confirm that TGF-beta1 or RA alone treatments derived cells differentiated into osteoblasts and were responsible for the bone that was deposited in ceramics, immunofluorescence staining to colocalize donor cells with osteoblasts-specific proteins was carried out. Tissue sections made from demineralized ceramics seeded with iPSC-cells derived by RA alone or in combination with TGF-beta1 were examined for osteocalcin and dentin matrix protein 1 (DMP-1) synthesis. Tissue sections made from mouse cortical bone were used as positive controls; ceramic scaffolds not seeded with cells were negative controls. Figure 5 illustrates immunofluorescence staining for osteocalcin in a murine cortical bone tissue section and in ceramics retrieved from recipient mice; FITC secondary antibodies were used for visualization (Figure 5). Figure 5A shows DAPI staining in a tissue section of a mouse cortical bone; Figure 5B is immunofluorescence staining for osteocalcin in the equivalent tissue section of the cortical bone. Immunofluorescence staining for osteocalcin is shown in green (Figure 5B). Figure 5C shows immunofluorescence staining in a tissue section in which primary antibody was omitted but processed similarly as in sections shown in Figure 5A and B (Figure 5). Figure 5D is DAPI staining of the tissue sections made from ceramics seeded with iPSC-TGF-beta1-RA-cells. Figure 5E is an imunofluorescence staining for osteocalcin in the ceramics seeded with iPSC-TGF-beta1-RA derived cells. Figure 5F is an overlay image of D and E indicating that cells seen in the ceramics synthesized osteocalcin thus confirming that the cells differentiated into osteoblasts *in vivo*. These data support the conclusion that iPSC-TGF-beta1-RA-cells differentiate into osteoblasts and deposit bone *in vivo*.

**Figure 6:**
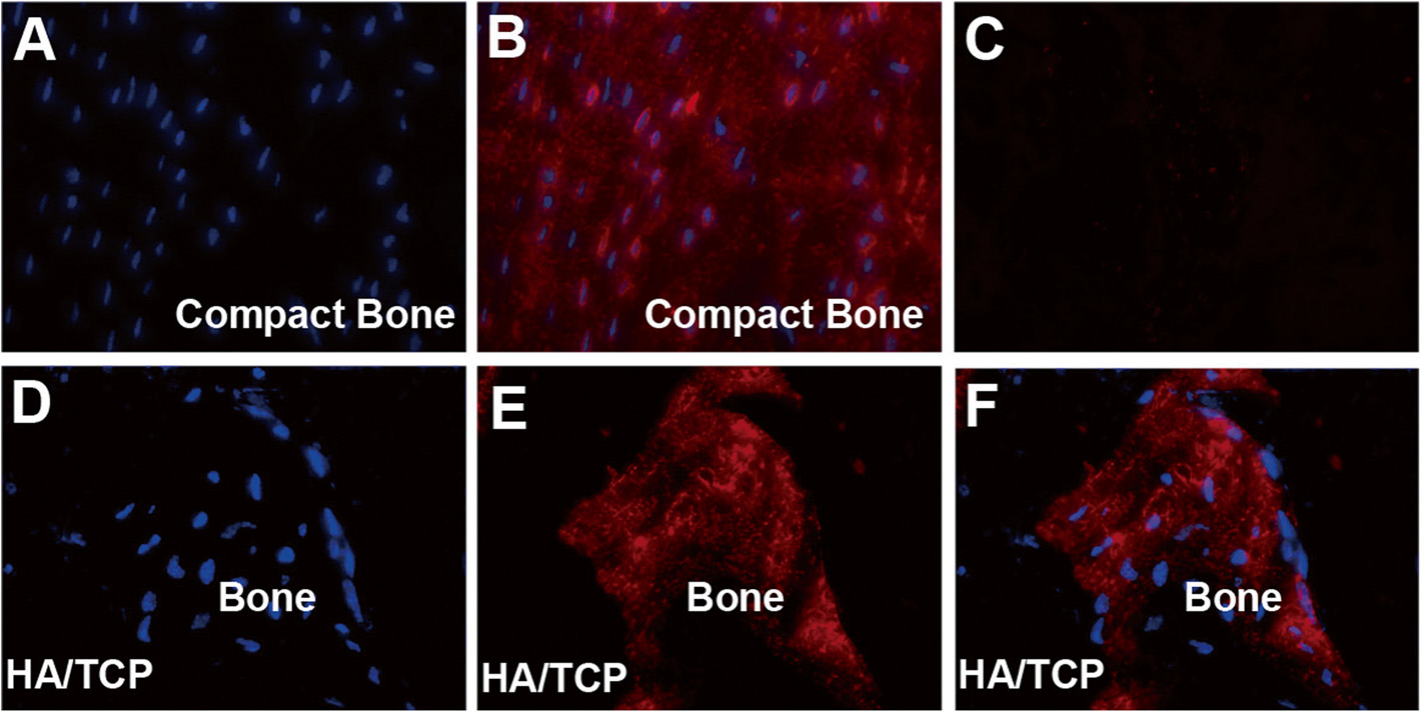
**Immunofluorescence staining for DMP1 in tissue sections of ceramic scaffolds seeded with iPSC-TGF-beta1-RA-cells and cortical bone.** (**A**) DAPI staining in a tissue section from a murine cortical bone; (**B**) Immunofluorescence staining for DMP1 in a tissue section of cortical bone; (**C**) Immunofluorescence staining in a tissue section in which primary antibody was omitted; (**D**) DAPI staining in a tissue section from a ceramic scaffold seeded with iPSC-TGF-beta1-RA-cells; (**E**) Immunofluorescence staining of DMP1 in a tissue section of a ceramic scaffold seeded with iPSC-TGF-beta1-RA-cells; (**F**) Overlay image of D and E indicating that DMP1 expressing cells colocalize with DAPI staining cells. Original magnifications 200X. The data confirm that iPSC-TGF-beta1-RA-cells proceed to osteocyte differentiation in ceramic scaffolds.

Dentin matrix protein 1 (DMP1) is an extracellular matrix protein synthesized by osteocytes [33,34]. We used immunofluorescence staining for DMP-1 to determine whether iPSC-derived cells proceeded to osteocytes differentiation in ceramics. Figure 6 shows the results of immunofluorescence staining for DMP1 in cortical bone and in ceramics seeded with iPSC-cells derived by TGF-beta1 treatment. Figure 6A is DAPI staining for cells in a tissue section made from a mouse compact bone which was used as a positive control; Figure 6B is an immunofluorescence staining for DMP1 in murine compact bone and Figure 6C is a control tissue section in which primary antibody was omitted. Figure 6D and E show DAPI and immunofluorescence staining for DMP1 in ceramics seeded with iPSC-TGF-beta1-RA-cells respectively. Figure 6F is an overlay image of 6D and 6E images. Because DMP1 is a secreted protein, DAPI staining in Figure 6D did not seem to align with DMP1 expressing cells in Figure 6E; nevertheless DMP1 staining was evident in the ceramics implanted with TGF-β1 iPSC-RA-cells. These data together with data presented in Figure 5, clearly confirm that iPSC-TGF-beta1-RA-derived cells differentiate into osteoblasts and osteocytes *in vivo* and deposit bone in ceramics implanted in SCID mice. Taken together, these data imply that TGF-beta 1 is the most effective in generating cells from iPSC with potential to differentiate into osteoblasts and deposit bone *in vivo* in comparison to other factors assessed in this study.

## Discussion

Induced pluripotent stem cells have generated excitement because of potential they possess for generating patient specific embryonic like stem cells [1-3,5]. There are many hurdles to overcome prior to clinical application of these cells; for example production of safer iPSC that would be suitable for clinical application and methods to direct them to specific lineages to avoid tumor formation. Some progress has been made toward generating safer iPSCs; for example, production of iPSC without use of viruses, use of protein and chemical factors to reprogram somatic cells [5-11]. In addition, efficiency in generating the cells has improved. Methods to direct iPSC or ESC to a limited number of specific lineages have also been reported [12-15], but this remains a challenge. Our interest is to direct murine iPSC to cells capable of differentiating into osteoblasts; this remains a challenging task [35,36]. Human and mouse iPSC and ESC display different responses to factors that control their differentiation to various cell lineages [37]. Differentiation protocols for human iPSC or ESC may not apply to murine cells. In the present report, we examined members of TGF-beta family for enhancing generation of cells that exhibit potential to give rise to osteoblasts and bone formation *in vivo*. The results showed that TGF-β1 and 3 in combination with RA were more effective in generating cells from iPSC with ability to give rise to osteoblasts and make bone in ceramics implanted in mice. Interestingly, although BMP-2 is a member of TGF-beta 1 family, iPSC-BMP-2-RA-derived cells exhibited a higher ability to adipocyte differentiation *in vitro*. These findings suggested that TGF-beta1 and BMP-2 display distinct activities toward iPSCs.

Several studies have demonstrated that BMPs play important roles in stem cell differentiation to various cell lineages [36,38,39]. BMP-2 has been shown to have a strong effect in inducing MSCs differentiation toward osteogenic and chondrogenic lineages under specific conditions [40]. Previous reports have shown evidence for participation of BMP2/4 in the commitment of pluripotent stem cells to the adipocyte lineage [41-44]. Specifically, exposure of BMP-2/BMP-4 to C3H10T1/2 cell line established from 14- to 17-day-old C3H mouse embryos induced these cells to commit toward adipocytic lineage [45-47]. These findings are in agreement with the present findings in which exposure of iPSC derived cells to BMP-2 induced their commitment to adipocyte differentiation. Members of the TGF-beta superfamily have been shown to play a role in induction of mesoderm in Xenopus, zebrafish, chicken and mouse [48,49]. TGF-beta1 was shown to promote early chondrogenesis during the embryonic endochondral ossification process [50]. Present findings suggest that TGF-beta1 may be playing a role of enhancing production of putative bone progenitors from iPSC at least in murine iPSC.

Few reports have shown bone formation *in vivo* by murine ESC-derived progenitors [30,51]. One report demonstrated that for murine ESC to make bone *in vivo* a cartilage template is required [30]. In another report RA was used to generate cells from murine iPSC that were shown to form calcified structures *in vitro* and *in vivo*[29]. The present report has shown that exposure of iPSC derived- EBs to TGF-β family of proteins in presence of RA enhanced production of cells with ability to differentiate toward osteoblastic lineage. In addition, TGF-beta1 derived cells formed larger bone surfaces than RA alone derived cells as determined by examination of tissue sections made from scaffolds seeded with RA alone and TGF-beta1-RA derived cells. The results suggest that use of TGF-beta-family of proteins may enhance generation of cells from iPS cells at least in murine that have potential to make bone *in vivo*. The data reported here showed that these cells were enriched in a population expressing CD73; in the same cell population, 10% of the cells expressed CD105. Expression of low CD105 and CD90 by murine iPSC derived cells were also shown previously in cells derived by RA treatment alone [29]. The relationship of these cells to MSCs is not clear because there is no one specific marker for identifying MSCs. Previous reports on generating cells with MSCs characteristics from hESC have used CD73 antigen to sort for cells with MSCs characteristics. The results showed that cells sorted based on this antigen were capable of giving rise to osteoblasts, adipocytes and chondrocytes *in vitro*[32]. Because these were human cells, these data cannot be extrapolated to murine cells.

## Conclusion

Taken together, the present findings showed that iPSC-derived cells by exposure of iPSC to TGF-beta1 or 3 in presence of RA enhances production of cells with ability to give rise to cells that deposit bone in ceramic scaffolds implanted in SCID mice. The results suggest that TGF-beta1 or 3 in presence of RA may be used to generate progenitors from murine iPSC that have potential to make bone. Although these results are of interest, they may not be applicable to human iPSCs. The data are however, of interest because they provide opportunities for investigating application of iPSCs in bone regeneration using murine models.

## Methods

### Generation of murine induced pluripotent stem cells (iPSC)

Stocks of murine iPSC generated previously by reprogramming tail tip fibroblasts (TTF) were used in the present studies. Methods for their production and characterization were described previously [31]. The iPSC generated from TTF were cultured on irradiated murine embryonic fibroblasts (MEFs) feeders in standard ES medium as described [31].

### Embryoid body formation (EBs) and generation of MSCs-like cells

iPSC colonies were trypsinized and transferred to ultralow attachment culture dishes (Corning, Corning, NY) to generate embyroid bodies (EBs). The EBs were maintained in ES medium without leukemia inhibitory factor (LIF) for 3 days. After 3 days of suspension culture, EBs were incubated in ES culture medium supplemented with 40 ng/ml retinoic acid (RA) for two days followed by two day incubation in a medium supplemented with either 10 ng/ml TGF-beta1, 10 ng/ml TGF-β3, 10 ng/ml bFGF or100 ng/ml BMP-2 for 3 days. Following incubation in this medium, cells were transferred onto 0.1% gelatin-coated plates and incubated in DMEM supplemented with 10% FBS and 50 μg/ml of ascorbic acid. When the cells were near confluent, they were trypsinized and replated onto 0.1% gelatin-coated tissue culture dishes and incubated in the same medium as above. After two passages, the derived cells were used for FACS analysis and osteogenic and adipogenic differentiation *in vitro* and *in vivo*.

### FACS analysis

The iPSC-derived EBs differentiated by retinoic acid and or various growth factor treatments were harvested and incubated in a buffer containing antibodies to selected putative MSCs surface antigens. Antibodies used for FACS analysis were phycoerythrin (PE) conjugated to anti-CD13, anti-CD34, anti-CD44, anti-CD45, anti-CD73, anti-CD90, anti-CD117, and unconjugated antibodies against CD105 (BD Biosciences, San Diego, CA). Methods described previously were used for preparation of the cells for FACS analysis [52]. In brief, a total of 2 × 10^5^ cells from different treatments were resuspended in 200 μl of Dulbecco’s PBS containing 2% FBS and 0.01% NaN3 and incubated for 30 minutes at 4°C with phycoerythrin (PE)-conjugated antibodies to surface antigens for analysis. This was followed by followed by PE-conjugated secondary antibodies (Santa Cruz Biotechnology Inc., Santa Cruz, CA). The proper isotype-identical Igs served as controls. After staining, the cells were fixed in 2% paraformaldehyde, and quantitative FACS analysis was performed on a FACStar flow cytometer (BD Biosciences, San Diego, CA). Each sample was tested three times.

### Osteogenic differentiation and bone formation

The iPSC-derived cells by different growth factor treatments were trypsinized, plated in six-well plates, and cultured in an osteogenic medium as described previously [53,54]. After 28 days of incubation, cells were stained in Alizarin Red S solution and examined under a light microscope. Alizarin deposits were extracted with 10% acetic acid and used for quantification of mineralization.

### Adipogenic differentiation

For adipogenic differentiation, iPSC-derived cells were plated in 12 well plates in adipogenic medium at a cell density of 5 × 10^3^ cells per well. The adipogenic medium was composed of DMEM with high glucose supplemented with 10% FBS, 0.1 mM indomethacin, 0.5 mM isobutylmethylxanthine (Sigma-Aldrich), and 10^-6^ M dexamethasone. The media were replaced every 3 days for 28 days. Adipogenic differentiation was assessed by Oil Red O staining at 3 weeks after initial adipogenic induction. For Oil Red O staining, the cells were rinsed in PBS and fixed in 10% formalin followed by incubation of the cells in 2% (wt/vol) Oil Red O reagent for 5 minutes at room temperature, examined under light microscope and photographed. The cells were suspended in 0.5 ml of isopropanol to extract Oil Red O for quantification of the level of adipogenic differentiation.

### *In vivo* bone formation

The Institutional Animal Care and Use Committee of Penn State University College of Medicine approved all animal procedures; all animal experiments were carried out following the approved protocol. Retinoic acid alone or in combination with TGF-beta1 iPSC derived cells were trypsinized and seeded onto HA/TCP ceramic scaffolds at 5 × 10^6^ cells/mL. Cells were allowed to attach to the ceramics for 2 h at 37°C prior to implantation in animals. Cell seeded Scaffolds were implanted subcutaneously onto the backs of thymic SCID mice. Five weeks after implantation; animals were sacrificed and the scaffolds were harvested.

### Histological analysis

For histological analysis, methods described previously were used [53]. Briefly, HA/TCP ceramic scaffolds seeded or not seeded with iPSC-derived cells and retrieved from recipient mice were fixed in freshly prepared 4% paraformaldehyde in PBS, containing 10% sucrose. Following fixation, the scaffolds were decalcified and embedded in paraffin. Ten micron tissue sections were cut, prepared for histological analysis and stained with H and E. Tissue sections were also stained by a modified Masson Trichrome Staining to demonstrate collagen synthesis [55].

### Immunofluorescence for Osteocalcin and Dentin matrix protein

Cryosections prepared from the ceramic scaffolds seeded or not seeded with cells were retrieved from recipient mice at 5 weeks following implantation. Tissue sections were fixed in cold acetone for 5 minutes and treated with 10% goat serum, followed by treatment with a polyclonal antibody specific for Osteocalcin (1:20 Millipore) or DMP-1 (1:50 RDI). Tissue sections made from murine cortical bone were treated similarly. For visualization, sections were treated with the secondary rabbit anti-rat antibodies conjugated with either FITC (Millipore) or Rhodamine (Santa Cruz, Santa Cruz CA) at a concentration of 1:1000 and 1:500 respectively.

### Gene expression analysis

Gene expression analysis of osteogenic and adipogenic associated genes were performed as described previously [53]. Triplicate PCR reactions were carried out.

### Statistical analysis

Statistical analysis was carried out using SPSS® software (SPSS, Chicago, IL). One-way ANOVA with a Tukey’s post-hoc analysis was used to evaluate for differences in growth factors treated and untreated samples for osteogenic and adipogenic differentiation and marker expression. Significance was set at P < 0.05.

## Competing interests

The authors declare that they have no competing interests.

## Authors’ contributions

Dr. FL performed the experiments and assisted in data interpretation and manuscript preparation. Dr. CN formulated the project idea, interpreted data and assisted in manuscript preparation and submission. All authors read and approved the final manuscript.
